# The genetics of chronic obstructive pulmonary disease

**DOI:** 10.1186/1465-9921-7-130

**Published:** 2006-10-20

**Authors:** Alice M Wood, Robert A Stockley

**Affiliations:** 1 Department of Medical Sciences, University of Birmingham, Birmingham, UK; 2 Lung Investigation Unit, University Hospitals Birmingham, Birmingham, B15 2TH, UK

## Abstract

Chronic obstructive pulmonary disease (COPD) is a heterogeneous disease caused by the interaction of genetic susceptibility and environmental influences. There is increasing evidence that genes link to disease pathogenesis and heterogeneity by causing variation in protease anti-protease systems, defence against oxidative stress and inflammation. The main methods of genomic research for complex disease traits are described, together with the genes implicated in COPD thus far, their roles in disease causation and the future for this area of investigation.

## Background

Chronic obstructive pulmonary disease (COPD) is characterised by airflow limitation that is not fully reversible, which usually progresses, together with an abnormal inflammatory response to noxious particles or gases [1]. Patients may have chronic bronchitis [2], emphysema[3], small airways disease or a combination of these, with or without systemic manifestations of the disease [4]. This results in great variety within the patient population. It is not yet clear what the significance of each disease component is in terms of cause, or effect on management, though research into genetics and pathogenesis is starting to clarify this.

Although cigarette smoking is the main environmental risk factor for developing COPD, only about 15% of smokers develop clinically significant disease [5], suggesting that there are other influences on disease expression. Previous studies estimated that smoking contributes 15% to the variability of lung function[6], whilst genetic factors account for a further 40%[7]. Family based studies support this: they have shown ancestral aggregation of spirometric measures in groups unselected for respiratory disease [8,9], and higher rates of airflow obstruction in first-degree relatives of patients with COPD[10]. Moreover, the observation of differences in rate of decline of lung function between smokers[11] suggests an interaction between genetic and environmental influences.

A genotype-environment interaction is defined by a non-additive contribution of gene and environment to the clinical phenotype[12]. Thus the two influences together confer a different level of risk than that expected by simply adding them. In a complex disease such as COPD there are likely to be many genes contributing to the overall phenotype, which may have additive or synergistic effects; these are known as epistatic interactions. When interpreting the results of genetic studies in complex diseases it is important to take such effects into account, lest a disease causing locus be missed. There are a variety of statistical methods that can allow for, detect or control for the presence of epistasis [13,14].

There are three main themes within the pathogenesis of COPD. The protease-anti-protease theory suggests that there is an imbalance between proteases that digest elastin, together with other components of the extra-cellular matrix, and anti-proteases that protect against this[15,16]. The origin of this theory was the observation that patients with α1-antitrypsin (an anti-protease) deficiency (AATD) develop early onset emphysema [17] implicating a role for its target enzymes (neutrophil elastase and proteinase 3), which can induce many of the features of COPD in animal models[18]. Subsequent work has suggested other important proteases, such as the matrix metalloproteinases (MMP's) [16], cathepsin B and collagenases [19] may also play a role, perhaps as part of a protease/anti-protease cascade.

**Figure 1:**
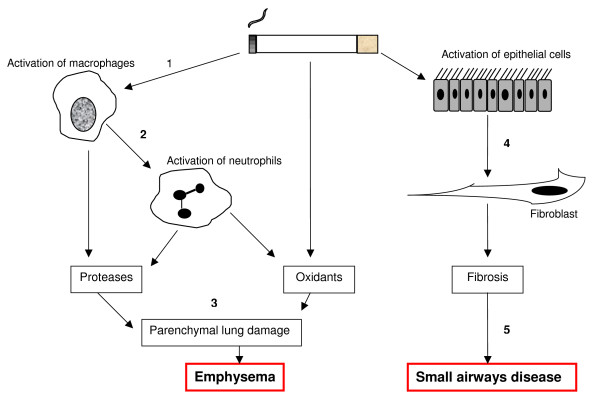
**The pathogenesis of COPD**. Cigarette smoke activates macrophages (1), leading to the direct release of proteases or neutrophil chemotracctants (2), together with the release of oxidants resulting in subsequent breakdown of connective tissue in the lung (3), causing emphysema. Epithelial cell stimulation promotes fibroblast activity (4), eventually leading to small airways disease (5).

The oxidant-antioxidant theory states that disparity between levels of harmful oxidants and protective antioxidants leads to oxidative stress, which in turn influences the actions of anti-proteases, and expression of proinflammatory mediators[20]. Both of these theories link to the third observation: the importance of inflammation in the pathogenesis of COPD[21]. These concepts are illustrated in Figure 1.

Polymorphisms in genes relating to proteases, antioxidants and inflammation have been found that relate to the presence of features of COPD, suggesting reasons for the heterogeneity of the observed clinical phenotype. This review will describe some of the methods that have identified candidate genes and summarise the evidence for a genetic basis to  COPD (see Table 2).

### How to identify candidate genes

Candidate genes may be suggested by pathogenesis, or vice versa. Variation, or polymorphism, within the gene can be classified in different ways[22], such as the structural nature of the change in the DNA, or its effect on the protein it codes for. Two common structural changes are microsatellites – multiple repeats of a short segment of DNA, and single nucleotide polymorphisms (SNP's) – a change of a single base. The latter are the most common type of polymorphism in the human genome[23]. Such changes may occur in coding regions of DNA (those that contribute to the making of a protein) or non-coding regions. If a change occurs in a coding region it can be described as non-synonymous or synonymous, depending on whether it affects the amino acid sequence of the gene product or not. Generally speaking non-synonymous changes in coding regions are more likely to alter the function of a protein[24], and hence to be related to disease. International projects, such as the SNP consortium[25], which catalogues common SNP's in the human genome, and HapMap[26,27], which has genotyped SNP's in 4 major ethnic groups, have contributed to the many databases available on genetic variation. Researchers can use such resources to identify potential disease causing polymorphisms, and their likely population frequencies, allowing the design of case-control association studies, looking for the polymorphism in those with and without the disease. This is a widely used approach, though often producing inconsistent results [28]; this may be because of variation in the definition of cases and controls, underpowered studies, racial differences and population heterogeneity. The issue of power is particularly important when examining a complex disease such as COPD, as each gene may contribute only a small amount to the clinical phenotype: if this results in a genotype relative risk of developing the trait of less than 2, then adequate power may not be achievable[29].

Linkage studies look for haplotypes, or short segments of the genome, conserved between generations by virtue of their size [30] – anything larger has the potential to be changed by recombination during meiosis. If a haplotype can be found that is passed down through a family, alongside a disease, then it suggests that there is a gene within or close to it that may have a functional effect on the disease. Haplotype analyses can also be useful in association studies, though difficult to perform[31]. This is because they allow for the possibility that a combination of SNP's within a gene may be causing the trait in question, rather than one of the SNP's alone[32].

Linkage is usually reported as a logarithm of the odds (LOD) score[33], which is a form of likelihood ratio derived from the recombination fraction between the marker and the proposed locus of the disease-causing gene. The threshold level of LOD score needed for genome-wide significance at 5% (p = 0.05) varies dependent on the study design, from 3.3 for family studies, using a proposed mode of inheritance (parametric linkage analysis) to 3.6 in sibling pair (non-parametric, no model of inheritance) studies [34]. Linkage analyses need to be followed by case-control association analyses for any genes in the area of interest that have a plausible link to disease – established because of potential in pathogenesis, or differential expression in the target tissue [35].

### Areas of interest from linkage studies

The major linkage study in COPD is the Boston early-onset COPD cohort [36-39], which performed genomewide linkage analysis in 585 members of families with early onset COPD, looking for linkage to pulmonary function test results. Areas of linkage were found for FEV1/FVC on chromosome 2, chromosome 1 and chromosome 17. The area on chromosome 2 was subsequently investigated by the same group, identifying *SERPINE2 *as a potential candidate gene. This area also contains the gene for the IL8 receptor, which might contribute to COPD since IL8 is involved in neutrophil chemotaxis to the lung [40](a critical process in delivery of destructive proteases). FEV1 linked to chromosomes 12 and 19 [38] in areas containing the genes for microsomal GST1 and TGFβ respectively. Mid expiratory flow, which is reduced in small airway disease in COPD, linked to chromosomes 2 and 12, together with a broader area on chromosome 19 [39].

### Proteases and anti-proteases

There are three classes of protease that have been studied in COPD – the serine proteases, which includes neutrophil elastase (NE)and proteinase 3, the cysteine proteases, such as cathepsin-B, and the matrix metalloproteases (MMP's) [41]. In general the serine proteases are capable of degrading elastin and some forms of collagen [41], whilst the MMP's have more of an effect on collagen, gelatin and laminin [16]. Each enzyme is inhibited by one or more anti-proteases, may inactivate other anti-proteases, or activate pro-inflammatory cytokines, such as TNFα, by interacting with proteinase activated receptors (PARs) [42]. The proteases function to clear debris and damaged tissue, but if their action is not effectively controlled they may produce excessive lung damage. The relationships between these enzymes, their inhibitors and some inflammatory mediators are shown in Table 1.

**Table 1:** Protease-antiprotease interactions

**Proteinase**	**Class**	**Activity**	**Active antiproteases**
Neutrophil elastase	Serine	Degrades elastin, collagen type IV & laminin	AAT
		Inactivates TIMP	SERPINA3
		Activates MMP9	SLPI
Cathepsin G	Serine	Degrades elastin, collagen I, II, IV & laminin	SERPINA3
		Activates MMP9	SLPI
Proteinase 3	Serine	Degrades elastin & collagen IV	AAT
		Activates TNFα	
Cathepsin B	Cysteine	Degrades elastin	Cystatin C
		Inactivates secretory leukocyte proteinase inhibitor (SLPI)	
MMP1	MMP	Degrades collagens I-IV, VII, VIII, X, XI	TIMP1-4
		Inactivates AAT	
		Activates TNFα	
MMP9	MMP	Degrades collagen IV, V, X, XIV & elastin	TIMP1-4
		Inactivates AAT	
		Activates TNFα & TGFβ	
MMP12	MMP	Degrades collagen I, IV, elastin & fibrillin	TIMP1-4
		Inactivates AAT	
		Activates TNFα	

#### AATD

AAT is an antiprotease that irreversibly inhibits NE, cathepsin G and proteinase 3. The AAT-NE complex also binds to receptors on neutrophils, thus stimulating further neutrophil migration, and amplifying inflammation. Its main function is to protect the connective tissue from NE released by activated neutrophils. There are four main variants of AAT, traditionally classified by their speed of movement during gel electrophoresis (F = fast, M = medium, S = slow, Z = very slow) [43], which are inherited in a co-dominant fashion. The PiM allele is the wild-type, and is the most prevalent. The PiZ allele is a more common deficiency variant in Northern Europeans, whilst the PiS deficiency variant is more common in South-West Europe [44]. AATD is classified by genotype and by the plasma AAT level. The PiZ variant is associated with significant AAT deficiency, lung and liver disease, though there is considerable disparity in clinical phenotype, which has been reviewed elsewhere [45].

The gene for AAT is on chromosome 14, and is highly pleomorphic. In addition to the common variants described here, there are over 100 SNP's catalogued in public databases [46]. Combinations of such SNP's, which give rise to six new haplotypes, have been associated with a higher risk of developing COPD in subjects without AATD [47]. However, even in patients with the same AAT genotype the phenotypes differ, suggesting that there may be other genetic modifiers present. One way to prove that modifier genes affect a complex disease is to show that traits related to the disease aggregate in families. In AATD the evidence so far is limited. Silverman et al showed some clustering of spirometric parameters in 82 families with PiZ or MZ genotypes, though this did not reach significance when adjusted for smoking status [48].

Case-control genetic studies have not been carried out as frequently in AATD as in usual COPD. One research paper examined polymorphisms in the gene coding for endothelial nitric oxide synthase (NOS) 3, and found a significant correlation between a SNP and severity of lung disease, defined by FEV1 [49]. NOS3 generates nitric oxide and citrulline from the amino acid arginine, as do the other isoforms of NOS [50]. The roles of nitric oxide (NO) in the lung include regulation of vascular tone and inhibition of inflammatory events, such as leukocyte adhesion; this has been reviewed extensively elsewhere [50]. It might therefore be expected that variation in the pathways that generate NO would have an impact on lung disease. In COPD, whether related to AATD or not, this could conceivably be due to alterations in the inhibition of inflammation. However the authors were unable to show any functional variation in NOS3 with this SNP, and concluded that it must lie in linkage disequilibrium with the gene that caused the association. Other family and case-control studies are underway and may begin to clarify reasons for phenotypic heterogeneity in AATD.

#### MMP's

The actions of MMP's include degradation of collagen, inactivation of AAT and activation of TNFα. Their action is reduced by tissue inhibitors of metalloproteinases (TIMP's). Studies using knockout mouse models have supported a role for MMP's in COPD. Mice over-expressing MMP1 develop emphysema [51], whilst those deficient in MMP12 are relatively protected [52]. Further support comes from clinical studies showing increased concentrations of MMP's in the bronchoalveolar lavage fluid of COPD patients [53].

The most widely studied MMP gene polymorphism is in the MMP9 gene, located on chromosome 20. A SNP in the promoter region (C→T, position -1562), which increases its activity has been described [54], and linked to COPD in both Chinese [55] and Japanese populations [56]. The Chinese cases were defined by airflow obstruction, according to the GOLD criteria, whilst the Japanese cases were defined by the degree of emphysema on CT scan. This latter group found that airflow obstruction did not correlate with presence of the T allele, though gas transfer corrected for alveolar volume (KCO) did. A later study has narrowed the emphysema phenotype linked to this polymorphism to upper zone predominant disease [57].

SNP's in *MMP1 *and *MMP12 *have also been studied in COPD. An insertion in the promoter region of *MMP1 *(G→GG, position -1607) that increases its transcription [58] by creating an extra transcription factor binding site has been described. This SNP occurs in 30% of the general population [58] and was negatively associated with rapid decline of lung function (defined by FEV1) in one case-control study[59]. This does not, however, have an explanation from its function. It would be expected that the GG variant would be positively associated if the higher level of *MMP1 *transcription lead to more lung damage. A role for polymorphisms of *MMP12 *was investigated by the same group, but an association with declining lung function was not seen [59]. A haplotype containing the *MMP1 *G→GG SNP, together with an *MMP12 *SNP that results in a change in protein composition (Asn357Ser), was found more commonly in the rapid declining group[59]. The authors suggested that this may be because the gene actually responsible for rapid decline lies in linkage disequilibrium with these two SNP's.

#### TIMP2

There are four TIMP's (TIMP1-4) that inhibit active forms of MMP. Although all TIMP's are capable of inhibiting any MMP their affinity for each MMP varies and TIMP2 has been shown to have a greater affinity for MMP2 and MMP9[60]. The contribution of various MMP's, TIMP1 and TIMP2 to emphysema have been investigated, and a key role for the MMP2-TIMP2 system proposed [61]. Two SNP's in *TIMP2 *are more common in Japanese subjects with COPD. One in the promoter region (that may cause reduced TIMP2 levels) and a second synonymous change in exon 3 [62]. This result has not yet been reproduced in other ethnic groups, and the functional consequences of each SNP remain theoretical, rather than proven. This should be an area for future research.

#### α-1 antichymotrypsin

α-1 antichymotrypsin (SERPINA3) inhibits cathepsin G and mast cell chymase in a reversible fashion. Two SNPs in *SERPINA3*, associated with low SERPINA3 levels, have been associated with COPD in Swedish subjects [63,64], though their cases were defined by a measure of airway resistance, rather than standard spirometric parameters. The positive results for these SNP's were not reproduced in Japan in patients with airflow obstruction and low FEV1 [65,66], though a non-synonymous mutation affecting the signal peptide region was found more commonly in the COPD group. All 3 of these mutations were examined in an Italian study of patients with airflow obstruction and were not found to be associated with disease, though their cases included subjects with bronchiectasis as well as COPD [66]. The variation in results between the studies could be explained by the different diagnostic criteria used by each group; it may be that the mutations are linked to airway resistance, but not to airflow obstruction, perhaps emphasising the heterogeneity of COPD.

### Antioxidants

**Figure 2:**
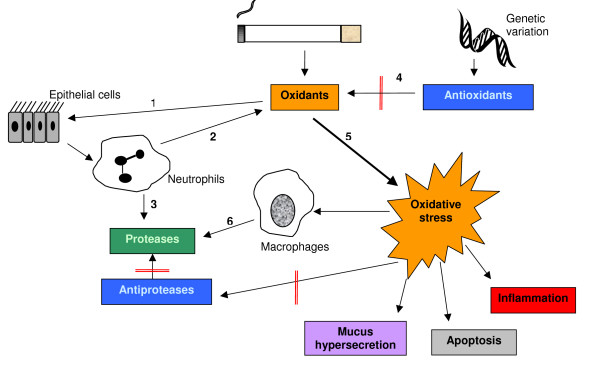
**Oxidative stress and its effects**. Oxidants contained within cigarette smoke irritate epithelial cells (1), releasing activating cytokines that prompt the recruitment of neutrophils and the release of cell derived oxidants (2) and proteases (3). Antioxidants inhibit oxidant mediated damage to the lung (4), but when an imbalance arises (perhaps because of gene polymorphisms) oxidative stress results (5). The consequences of oxidative stress include activation of macrophages (6), leading to the production of more proteases, mucus hypersecretion, epithelial cell apoptosis, inflammation and inhibition of the action of antiproteases.

Oxidative stress results from an imbalance between exogenous, harmful, oxidants and endogenous, protective, antioxidants[20]. This process, illustrated in Figure 2, can damage components of the lung matrix (such as elastin), injure the airway epithelium and enhance inflammation in the lung via up-regulation of genes for pro-inflammatory cytokines[20]. Cigarette smoke is a major source of oxidants (mainly free radicals and nitric oxide). Oxygen radicals are also released by inflammatory leukocytes, which are known to accumulate in the lungs of smokers[67], thus exacerbating the process of oxidative damage. Antioxidant enzymes present in the airway include glutathione-S-transferase, superoxide dismutase and catalase[68], amongst others. Gene polymorphisms affecting the function of such proteins might alter the amount of oxidative stress and so have been examined for their link to COPD.

#### Glutathione-S-transferases

The glutathione-S- transferase (GST) genes code for a family of enzymes that detoxify some of the harmful contents of tobacco smoke [69]. Polymorphisms in the genes are known to have functional consequences, and have been examined in COPD [70-72]. The two variants with the most evidence supporting a role in the disease are *GSTP1 *and *GSTM1*.

*GSTP1 *contains two known SNP's, though only one is known to have an effect on the catalytic activity of the enzyme. This is an A→G change at nucleotide +313, resulting in a single amino acid substitution (Ile105Val) [73] shown to increase the metabolism of carcinogenic aromatic epoxides [74]. Studies of the relationship of this variant to lung disease have varied in their results. It would be expected that the 105Ile variant would be associated with higher levels of lung damage, since it is less active against oxidants – this was confirmed by an association with airflow obstruction in a Japanese population [71]and replicated in a Caucasian population in the Lung Health Study (LHS), where this polymorphism together with a family history of COPD was linked to rapid decline of FEV1 (OR = 2.20, p = 0.01)[70]. Conversely the same group showed that the 105Val variant was associated with low baseline lung function (OR = 1.69, p = 0.016) and rapid decline in the higher baseline group (p = 0.017) [72], whilst Gilliland [75]demonstrated reduced annual growth rates for FEV1 and FVC in children homozygous for the 105Val variant. The latter results are difficult to explain on the basis of this gene's action alone, but might be understandable if there are gene-smoking or gene-gene interactions affecting the expression of the gene product. No gene-smoking affects were seen in the LHS [72], but there may be an additive effect of polymorphisms in *GSTP1 *and other GST genes[70], suggesting that a consequence might not be seen unless a change in several gene products were present.

*GSTM1 *has 3 known alleles, one of which is a null allele, such that homozygotes for the null allele have no detectable GSTM1 activity. This genotype has been associated with emphysema [76] and chronic bronchitis [77], with conflicting results concerning its role in lung cancer [76,78]. In common with most other genetic studies in COPD the positive results have been difficult to replicate [28], though this may be because studies looked at different subgroups of patients with COPD. The negative studies defined their cases by airflow obstruction [79] and rapid decline in FEV1 [70], hence might not have picked up a change in gene prevalence in chronic bronchitics. This difference in case definition remains a common theme in COPD genetics studies.

#### Superoxide dismutase

There are three superoxide genes, coding for scavengers of reactive oxygen species (ROS) [68]. Extracellular superoxide dismutase (SOD3) is present at high concentrations in areas of the lung containing large amounts of type 1 collagen, especially around large airways and also adjacent to alveoli [80]. It is thought to have a role in protecting the lung, particularly during inflammation [81,82]. A SNP (C→G substitution at +760) of *SOD3 *that increases plasma enzyme levels has been examined in 2 studies relating to COPD [68,83], and found to have a protective effect. A case-control study was carried out in New Zealand, where the mutation was found more frequently in resistant smokers than in those with COPD(OR = 4.3, p < 0.05) [68]. The second study was part of the Copenhagen City Heart Study, which examined 9258 individuals in both cross-sectional and prospective study designs [83]. This demonstrated a reduced risk of developing COPD in smokers (OR = 0.4) and a reduced risk of hospital admission or death due to COPD (hazard ratio = 0.3) in those carrying the mutation. Since this effect was not seen in non-smokers, whose odds ratio of developing COPD when they carried the mutation was 1.5, it suggests a gene-smoking interaction, though this could not be statistically proven.

#### Microsomal epoxide hydrolase

Microsomal epoxide hydrolase (EPHX1) is expressed in bronchial epithelial cells and metabolises highly reactive epoxide intermediates in cigarette smoke [84,85]. There are 2 known SNP's in this gene that affect enzyme activity by a single amino acid substitution. The first SNP is in exon 3 (Tyr113His), the second in exon 4 results in a further change in protein constitution (His139Arg). In both cases the His variant is associated with lower levels of enzyme activity [86,87]. Both polymorphisms only account for a modest change in activity level [87], so it may be that there is also variation in the gene's regulatory regions [88].

Patients carrying both His variants were at the highest risk of developing COPD (OR = 4.1, p < 0.001) and emphysema (OR = 5, p < 0.001) in a Scottish population[89]. This result was replicated in those with more advanced COPD in Japan (OR = 2.9, p = 0.02)[90] despite the differing frequency of genotypes between the two racial groups. The LHS demonstrated a relationship with rapid decline in lung function (FEV1) for the same haplotype, though this was only statistically significant for those with a family history of COPD [91]. The His139 variant alone was associated with a spirometric diagnosis of COPD in the Boston early-onset COPD cohort [28].

The contribution of this gene to the heterogeneity of COPD has been examined in more detail in the National Emphysema Treatment Trial (NETT) Genetics Ancillary Study [92]. The authors studied a number of polymorphisms and looked for correlation between genotype and functional capacity phenotypes in two separate patient groups, hypothesising that there is a genetic basis to the observed phenotypes. The exon 3 SNP (Tyr113His) was associated with poor exercise capacity, whilst the exon 4 SNP (His139Arg) was connected to relatively greater gas transfer (DLCO). This study was powered to detect a moderate effect of each genotype on overall phenotype, so taken with the previous positive studies it seems likely that these polymorphisms contribute to the COPD phenotype. Their link to specific subgroups of COPD patients will need further study.

#### Heme oxygenase-1

Heme oxygenase-1 (HMOX1) is an enzyme important in heme metabolism, which catalyses the oxidative cleavage of heme, resulting in the release of carbon monoxide, bilverdin and iron [93]. Bilverdin is then broken down into bilirubin, which scavenges local ROS; thus HMOX1 contributes to the generation of antioxidants. It is present at higher concentrations in the lungs of smokers than non-smokers, suggesting up-regulation in these circumstances [94], presumably because of a response to increased ROS.

A microsatellite (GT)n repeat in the 5' region of *HMOX1 *has been described that seems to alter the level of transcription when under thermal stress [95]. When the microsatellite is longer, it is not induced as effectively by ROS [96]. This suggests that in the presence of a long GT repeat (for instance n ≥ 30) smokers would not be able to protect their lungs from the damage induced by ROS in smoke, and thus would be more susceptible to emphysema. Two clinical studies have shown a link between this *HMOX1 *polymorphism and COPD. A Japanese case-control study showed that patients with 30 or more GT repeats in the microsatellite region were more likely to have emphysema, diagnosed by CT scan[96]. A larger study in France showed that 33 or more GT repeats was associated with airflow obstruction and more rapid decline of lung function, particularly in smokers [97]. They were able to show a significant gene-smoking interaction (p = 0.0006 for FEV1/FVC decline). This effect on decline was not, however, reproduced in the LHS [70].

### Inflammation and inflammatory mediators

**Figure 3:**
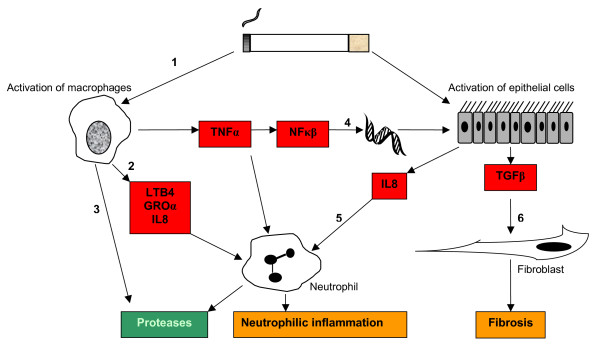
**Inflammatory mediators in COPD**. There are many pro-inflammatory mediators involved in COPD, some of which are illustrated here. Cigarette smoke activates macrophages (1) to release TNFα, LTB4, IL8 and other neutrophil chemotactic factors (2), as well as proteases (3). TNFα promotes further IL8 release from other cells in the respiratory tract by NFκβ mediated effects on gene transcription (4). This increases local neutrophilic inflammation (5), and hence the release of proteases. Epithelial cells also stimulate fibroblasts via TGFβ, leading to fibrosis (6). TNF = tumour necrosis factor alpha, LTB4 = leukotriene B4, IL8 = interleukin 8, GRO = growth related oncogene, TGF = transforming growth factor, NF = nuclear factor

It is generally accepted that COPD is associated with an abnormal inflammatory response [1]. This extends beyond the lung to systemic manifestations [98]. Many different mediators have been implicated in pathogenesis [99] and their roles are summarised in Figure 3.

#### TNFα

TNFα mediated inflammation is thought to play a key role in both the respiratory [100] and systemic features of COPD [98]. A SNP in the promoter region of the TNFα gene (G→A at position -308) directly affects gene regulation, and is associated with high TNFα production [101]. This polymorphism has been studied in several COPD related phenotypes, with differing results. An initial case-control study in Taiwan examined subjects with chronic bronchitis, hypothesising that this was linked to increased airway inflammation [102]. They found an increased prevalence of the polymorphism in cases relative to controls (p < 0.01, OR = 11.1). It has also been linked to airflow obstruction without chronic bronchitis, and severity of emphysema in Japanese subjects [103,104]. Studies in Caucasians have not been able to reproduce these results [28,91] which might be explained by variation in genotype frequencies between races (data available from HapMap[27]), or by linkage dysequilibirum with HLA alleles, seen previously in the Caucasian population [105].

#### TGFβ

TGFβ1 regulates extra-cellular matrix production, cell growth and differentiation, tissue repair and some immune responses [106]. Mice who are unable to activate latent TGFβ develop emphysema via alterations of MMP12, suggesting that disordered activation relates to the pathogenesis of COPD [107]. A linkage analysis in the Boston early-onset COPD study showed association between an area of chromosome 19 containing the TGFβ1 gene and FEV1[108]. Three SNP's in this gene had a significant association with severe COPD in the NETT cohort [108]. This association was replicated for two of the SNP's by Hersh et al[92], who linked them both to subjective measures of dyspnoea, though not objective measures of exercise capacity. This apparent discordance may be important when defining phenotypes within COPD.

The two SNP's identified by Hersh et al both have an effect on TGFβ1 levels. The first is a C→T change at position -509, in the promoter region, which enhances promoter function, thus increasing levels of TGFβ [109]. The second is a C→T change at position 613, which leads to an amino acid substitution (Leu→Pro) and higher production of TGFβ1 [110]. If both of these polymorphisms are implicated in COPD, it suggests that TGFβ may have a protective role. A case-control study examining the latter SNP in COPD subjects, resistant smokers and healthy controls concurred, finding that the Pro allele was less common in COPD subjects relative to resistant smokers (OR = 0.59, p = 0.01) and controls (OR = 0.62, p = 0.005). Further research on the role of TGFβ in COPD may help to clarify if this association has credibility in relevant pathogenic processes.

#### Vitamin D binding protein

Vitamin D binding protein, also known as Gc globulin, is a precursor of macrophage activating factor (MAF) [111] and enhances the neutrophil chemotactic properties of C5 derived peptides [112]. The latter function is prevented by neutrophil elastase inhibitors [113], suggesting a relationship between the protease-antiprotease pathway and inflammation. This would fit well with a role for vitamin D binding protein in the pathogenesis of COPD.

A number of studies have looked for links between polymorphisms in this gene (*GC*) and COPD. Two non-synonymous SNP's have been identified, which represent the *GC2 *and *GC1S *alleles. The *GC2 *allele has been found to be protective in studies of Caucasian subjects [114,115], consistent with the fact that only 10% of this form can be converted to MAF [116]. No role has been proven for this allele in neutrophil chemotaxis [114]. The *GC1S *allele has not been shown to have a significant association with COPD [28]. In Japanese subjects the *GC1F *allele has been linked to an increased risk of developing airflow obstruction, emphysema and a rapid decline of FEV1 [117,118]. Caucasian patients homozygous for this allele were at increased risk of developing COPD in one study [115] but not in another [114]. Neither could the link to rapid decline be reproduced in this racial group [91]. The difference in allele frequency between racial groups may explain why studies in Caucasians (who have a lower frequency of the 1F allele) have been unable to detect an association, as they would have required greater patient numbers to be adequately powered. An alternative explanation is that there is racial variation in gene associations with COPD.

#### IL13

Studies in transgenic mice have shown that if IL13 is over expressed, it results in cathepsin and matrix metalloproteinase dependent emphysema with mucus metaplasia [119]. A polymorphism in the promoter region (C→T, position -1055) is associated with increased IL13 production [120], with the T genotype being more common in COPD patients [121]. In mice IL13 induced emphysema is characterised by excessive pulmonary mucus production, so further studies looking for the prevalence of this polymorphism in the subgroup of COPD patients with chronic bronchitis might be worthwhile.

### Gene products without an identified role in pathogenesis

#### Surfactant proteins

The surfactant proteins are hydrophobic proteins that contribute to regulation of surface tension in the alveoli. Components of surfactant also have a role in host defence and control of inflammation. Alterations of surfactant might therefore be a factor in COPD, as suggested by mathematical models of emphysema [122] although this has yet to be studied in vivo. A SNP in the gene coding for surfactant protein B (SFTPB), which causes a single amino acid substitution (Thr131Ile), has been associated with COPD in the Boston Early-onset COPD cohort [28], and in a case-control study in Mexico [123]. In the NETT cohort this was also seen when gene-environment interaction was taken into account, where it was associated with dyspnoea score and exercise capacity[92]. In the Mexican study a number of SNP's and microsatellites were examined, with mutations in *SFTPB *(or microsatellite markers linked to it) being the most closely associated with COPD.

#### SERPINE2

The SERPINE2 gene was identified as having a potential role in COPD by a novel method. Firstly linkage of airflow obstruction to an area on chromosome 2 in the Boston early-onset COPD cohort [37,38], followed by integration of these results with knowledge of genes expressed during murine lung development, together with human lung microarray datasets from control subjects and those with severe COPD [35]. Multiple SNP's in this gene were examined in patients from the NETT cohort, with several being significantly associated [35]. A subsequent large case-control study did not, however, find any association with COPD in European patients [124] and questioned the validity of some of the results reported in the original study.

SERPINE2 has not been studied in COPD. It is known to be an inhibitor of trypsin-like serine proteases, but not neutrophil elastase [125], which might have indicated a role in the protease-antiprotease pathways. Its major function is in coagulation and fibrinolysis [126]. Although enhanced prothrombotic markers have been linked to decline of FEV1 in one small study in COPD [127] this has not been widely investigated.

### The future

There are several areas in which methodology of genetic studies is advancing. Animal model genetics may help in clarifying some aspects of pathogenesis. One study has been performed which showed differences in inflammatory cell and cytokine profiles between murine strains after exposure to smoke[128]. If this type of study were followed by quantitative trait locus analysis it may help to identify candidate genes for further study in humans. Genome-wide association analysis may now be performed looking for up to 500000 SNP's at any one time to identify regions in linkage disequilibrium (LD) with features of COPD. This approach does, however, have limitations. Firstly, the SNP's should be as independent as possible from one another, to avoid the complication of LD between them. If this is not the case statistical corrections for multiple testing will not be valid, as the variables would be related. This means that haplotype tagged SNP's should be used, but even with these, the number needed to identify all common variants across the genome is uncertain with estimates ranging from 180000 to 600000 [129]. Secondly, statistical adjustments will be needed to account for multiple testing. Software to help with analysis of large genetic datasets is available from industry[130] and academia[131] and is necessary to handle the huge amounts of data that a genome-wide study would generate. Thirdly the potential costs of such studies could be prohibitive. Finally, the number of areas being investigated will raise the potential for false positive results, so confirmation of any positive results in multiple independent populations should be sought.

As more genes are identified we may be able to characterise patients with COPD more accurately and target therapies to those subgroups most likely to benefit.

## Competing interests

The author(s) declare that they have no competing interests.

## Authors' contributions

AMW drafted the manuscript. Both authors read and approved the final manuscript.

**Table 2:** Some genetic polymorphisms relevant in COPD

***Gene*/Protein**	**Polymorphism ID**	**Gene ID**	**Role**	**Function**
*MMP9*	CR994492*	4318	C-1562T	Increases promoter activity
*MMP1*	rs1799750	4312	G-1607GG	Increases transcription
MMP12		4321	Asn357Ser	
*TIMP2*	rs2277698	7077	G853A	
			G-418C	
SERPINA3	rs4934	12	Ala-15Thr	Alters signal peptide
	rs17473		Pro227Ala	Reduces enzyme level
	rs1800463		Leu55Pro	Reduces enzyme level
GSTP1	rs947894	2950	Ile105Val	Increased enzyme activity
GSTM1	CG931302*	2944	Null	No enzyme activity
SOD3	CM941295*	6649	Arg213Gly	Increases enzyme level
EPHX1	rs1051740	2052	Tyr213His	Reduces enzyme activity
	rs2234922		His139Arg	Increases enzyme activity
*HMOX1*	CE000297*	3162	(GT)_n_	Alters transcription
*TNFα*	rs1800629	7124	A-308G	Increases TNFα level
*TGFβ*	rs1800469	7040	C-509T	Increases TGFβ level
	rs1982073		C613T	Increases TGFβ level
GC	rs4588	2638	Thr436Lys	Decreases conversion to MAF
	rs7041		Asp432Glu	
*IL13*		3596	C-1055T	Increases IL13 production
SFTPB	rs1130866	6439	Thr131Ile	
*SFTPB*	D2S388**		(CA)_n_	

**Polymorphism ID: **if listed in Entrez SNP [132], the reference SNP (rs) number is given.

*indicates an accession number from the Human gene mutation database [133].

** is searchable from PubMed, but not Entrez SNP.

**Gene ID: **from Entrez Gene [134].

**Role **This indicates the alteration and location of the nucleotide polymorphism (e.g. G853A, meaning a G→A substitution at position +853) or shows the change that results in the amino acid sequence (e.g. Ile105Val, meaning a change from Ile→Val at position 105 within the protein). Microsatellites are denoted (xx).
